# Characterizing the Effect of the Lysine Deacetylation Modification on Enzyme Activity of Pyruvate Kinase I and Pathogenicity of *Vibrio alginolyticus*

**DOI:** 10.3389/fvets.2022.877067

**Published:** 2022-06-06

**Authors:** Zhou Xu, Linjing Wang, Xudong Wang, Mingyue Wan, Mei Tang, Yu Ding

**Affiliations:** ^1^Fisheries College, Guangdong Ocean University, Zhanjiang, China; ^2^Guangdong Provincial Key Laboratory of Pathogenic Biology and Epidemiology for Aquatic Economic Animals, Zhanjiang, China; ^3^Guangdong Key Laboratory of Control for Diseases of Aquatic Economic Animals, Zhanjiang, China

**Keywords:** *Vibrio alginolyticus*, lysine deacetylation, PykF, post-translational modification, glycolysis

## Abstract

Pyruvate kinase I (PykF) is one of the key enzymes of glycolysis and plays a crucial role in bacterial metabolism. Several acetylation sites of *Vibrio alginolyticus* PykF were reported in previous studies and then 11 sites were first verified in this study, however, the specific roles of PykF acetylation remains unclear. Overlap-PCR and homologous recombination were implied to delete *V. alginolyticus pykF* gene and constructed complementary strains of site-directed mutagenesis for the further research focus on the deacetylation regulation on PykF. The results showed that the pyruvate kinase activity was sharply suppressed in the deacetylation status of K52, K68, and K317 of PykF, as well as the extracellular protease activity was significantly decreased in the deacetylation status of K52 and K68, but not induced with K317. Moreover, the growth rates of *V. alginolyticus* were not influenced with these three deacetylation sites. The Δ*pykF* mutant exhibited a 6-fold reduction in virulence to zebrafish. Site-directed mutations of K52R and K68R also showed reduced virulence while mutations of K317R didn't. The *in vitro* experiments showed that PykF was acetylated by acetyl phosphate (AcP), with the increase of incubation time by AcP, the acetylation level of PykF increased while the enzyme activity of PykF decreased correspondingly. Besides, PykF was deacetylated by CobB deacetylase and in result that the deacetylation was significantly down-regulated while the pyruvate kinase activity of PykF increased. Moreover, deletion of *cobB* gene had no significant difference in pyruvate kinase activity. These results confirm that CobB can regulate the acetylation level and pyruvate kinase activity of PykF. In summary, the results of this study provide a theoretical basis for further understanding of the deacetylation modification of PykF. It provides a new idea for the prevention and cure of vibriosis.

## Introduction

*Vibrio alginolyticus* is an opportunistic halophilic gram-negative bacterium, which has great harm to the development of aquaculture and human health (1–4). *V. alginolyticus* is associated with diseases of fish, shellfish, shrimp, and coral (5–7), and is also a human pathogen that causes gastrointestinal infections (8). Therefore, it is necessary to understand the pathogenic mechanism of *V. alginolyticus* to cure these diseases. Lysine acetylation is a reversible post-translational modification (PTMs) that are involved various cellular processes in organisms from eucaryote to prokaryote (9–12). Lysine acetylation has a wide implication on bacteria, and plays an important role in their physiology, metabolism and virulence (13–15). In bacteria, it is known that protein activity is regulated by site-specific acetylation. In addition, acetyltransferases and deacetylases regulate the activity of proteins by adding or removing charged acetyl groups (14). In contrast to the transcriptional regulation, PTMs such as acetylation usually finetune the activities of proteins rather than turn them on or off (16, 17). In some pathogens, lysine acetylation modification affects bacterial virulence by regulating the activity of key factors, such as acetylation regulating protein stability and DNA-binding ability of HilD to modulate *Salmonella Typhimurium* virulence (18), acetylation of lysine 201 inhibits the DNA-binding ability of PhoP to regulate *Salmonella* virulence (19), acetylation of PhoP K88 is involved in regulating *Salmonella* virulence (20), acetylation is involved in *Salmonella enterica Serovar Typhimurium* Virulence and so on (15, 21). Furthermore, lysine acetylation can regulate the activity of various metabolic enzymes, such as glyceraldehyde phosphate dehydrogenase, isocitrate lyase, isocitrate dehydrogenase kinase/phosphatase, isocitrate dehydrogenase, malate dehydrogenase citrate synthase (22–25). This suggests that acetylation modifications may play an important role in bacterial. But no studies have been reported about PTMs of vibrio key factors/proteins regulate vibrio virulence. In past studies, acetylome and succinylome of vibrio have shown that a key strategy of vibrio is to use PTMs to modulate essential factors for virulence. These essential factors are involved purine metabolism, ribosome, pyruvate metabolism, glycolysis/gluconeogenesis, the TCA cycle, and so on, include LuxR (Transcriptional activator protein LuxR), LuxO (Luminescence regulatory protein LuxO), LuxS (S-ribosylhomocysteine lyase), Tdh (L-threonine 3-dehydrogenase), SodB (Superoxide dismutase), PEPCK [Phosphoenolpyruvate carboxykinase (ATP)], ClpP (ATP-dependent Clp protease proteolytic subunit) and so on, they are very important to virulence systems of vibrio (26–29). At present, how acetylation affects *V. alginolyticus* metabolism as well as virulence is still uncovered.

Previous studies about global acetylome studies have revealed that PykF is acetylated at multiple sites (27, 30–32). In the last step of glycolysis, pyruvate kinase catalyzes the irreversible transfer of phosphate from phosphoenolpyruvate (PEP) to ADP resulting in the production of pyruvic acid and ATP. In mammals, two genes, respectively, encode two different PK (pyruvate kinase) isoforms. *Pkrl* gene encodes the PKL and PKR isoforms of PK, which expresses in the liver and red blood cells, respectively. Most tissues express the PKM1or PKM2 isoform encoded by *Pkm* gene (muscle form of PK) (33). Acetylation at Lys305 of the PKM decreases its activity and targets it for chaperone-mediated autophagy and subsequent lysosome degradation (34). Two isoenzymes of PK, PykF and PykA (pyruvate kinase II), have been identified in *E. coli* (35, 36). Acetylome studies have revealed that *V. alginolyticus* PykF is acetylated at multiple sites, but the effect of their acetylation sites on its biological functions is still unknown. In this study, 11 lysine acetylation sites of *V. alginolyticus* PykF were identified by specific antibody enrichment combined with high-resolution mass spectrometry analysis. We mimicked the effect of lysine deacetylation of *V. alginolyticus* PykF on its biological function by site-directed mutagenesis. In summary, the results in this study provide a theoretical basis for further understanding of the acetylation modification of PykF.

## Materials and Methods

### Strains and Plasmids

*Vibrio alginolyticus* HY9901 was isolated from a diseased red snapper (*Lutjanus sanguineus*) in Zhanjiang Port, Guangdong Province (37). The strains and plasmids used in this study are in Table 1. All primers were designed as shown in Table 2 according to the *V. alginolyticus* gene sequence (GenBank Number: GU074526.1) and the *pykF* gene (GenBank Number: OK376642). Healthy zebrafish (*Danio rerio*) were purchased from Zhanjiang Aquatic Market, with an average body length of 4 cm and weight of 0.2 g. The zebrafish were performed by bacteriological recovery tests and temporary rearing in seawater in a circulation system at 28°C for 2 weeks before the experiment.

**Table 1:** Bacterial strains and plasmids used in this study.

**Strains, plasmids**	**Relevant characteristics**	**Source**
*V.alginolyticus* HY9901	Wild type, isolated from diseased *Lutjanus sanguineus* off the Southern China coast	(37)
*E. coli* BL21(DE3)	Expression vector, Kana	TransGen Biotech
S17-1 (λpir)	T prSmrrecA thi pro hsdR-M+RP4:2-Tc: Mu: K m T n7 λpir	In this lab
pLP12	suicide plasmid, Cmr	Guangzhou KnoGen Biotech
PMMB207	High copy plasmids, Amp^R^, Cm^R^	Hubei Bio Transduction Lab

**Table 2:** Sequences of primers used in this study.

**Primer name**	**Primer sequence (5^′^-3^′s^)**
*pykF*-F	CGCGGATCCATGAAAAAGACCAAAATCGTATG
*pykF*-R	CCGCTCGAGTTATAGTACGTGTACAGAAGCTG
*pykF*-MF1	GGAATCTAGACCTTGAGTCGGTTCATCAACGCTGACTTCTCC
*pykF*-MR1	CGCCAGAAACCATAACAACGATAGTGCCGTGTTCTTCGTAGTCA
*pykF*-MF2	TGACTACGAAGAACACGGCACTATCGTTGTTATGGTTTCTGGCG
*pykF*-MR2	ACAGCTAGCGACGATATGTCGCTTTCGCCAGGTTTTACTCG
pLP-UF	GACACAGTTGTAACTGGTCCA
pLP-UR	CAGGAACACTTAACGGCTGAC
*pykF*-TF	AATGATGCTGCTGCTTTTGCT
*pykF*-TR	GTTCCCTGTGCCTAAAATCTGC
T7-TER	TGCTAGTTATTGCTCAGCGG
T7T	TAATACGACTCACTATAGGG
*pykF*-K13Q-F	TGTACGATTGGCCCTCAAACAGAATCTGTAGAG
*pykF*-K13Q-R	CTCTACAGATTCTGTTTGAGGGCCAATCGTACA
*pykF*-K13R-F	TGTACGATTGGCCCTAGAACAGAATCTGTAGAG
*pykF*-K13R-R	CTCTACAGATTCTGTTCTAGGGCCAATCGTACA
*pykF*-K19Q-F	GAATCTGTAGAGCAGCTAACTGAACTAGTTAAC
*pykF*-K19Q-R	CTGCTCTACAGATTCTGTTTTAGGG
*pykF*-K19R-F	CAGAATCTGTAGAGAGGCTAACTGAA
*pykF*-K19R-R	CTCTCTACAGATTCTGTTTTAGGGC
*pykF*-K52Q-F	ATCGCGAACTTCCGTCAAGTAATGGAAGCTACT
*pykF*-K52Q-R	TTGACGGAAGTTCGCGATACGAGTG
*pykF*-K52R-F	TCGCGAACTTCCGTAGAGTAATGGAA
*pykF*-K52R-R	CTACGGAAGTTCGCGATACGAGTGCCG
*pykF*-K59Q-F	GAAGCTACTGGCCAACCACTAGCAATTCTTCT
*pykF*-K59Q-R	TTGGCCAGTAGCTTCCATTACTTTAC
*pykF*-K59R-F	TGGAAGCTACTGGCAGACCACTAGCA
*pykF*-K59R-R	CTGCCAGTAGCTTCCATTACTTTAC
*pykF*-K68Q-F	CTTCTAGATACTCAAGGTCCAGAAATCCGC
*pykF*-K68Q-R	TTGAGTATCTAGAAGAATTGCTAGTGG
*pykF*-K68R-F	TTCTTCTAGATACTAGAGGTCCAGAA
*pykF*-K68R-R	CTAGTATCTAGAAGAATTGCTAGTGG
*pykF*-K145Q-F	ACTGAAGTTAAATGTCAAGTTCTTAACAACGGT
*pykF*-K145Q-R	TTGACATTTAACTTCAGTTTCAGTCT
*pykF*-K145R-F	CTGAAGTTAAATGTAGAGTTCTTAAC
*pykF*-K145R-R	CTACATTTAACTTCAGTTTCAGTC
*pykF*-K317Q-F	GGTGAAACGGCGCAAGGTAAATACCCTGTT
*pykF*-K317Q-R	TTGCGCCGTTTCACCAGAAAGCATTAC
*pykF*-K317R-F	CTGGTGAAACGGCGAGAGGTAAATAC
*pykF*-K317R-R	CTCGCCGTTTCACCAGAAAGCATTA
*pykF*-K319Q-F	GAAACGGCGAAAGGTCAATACCCTGTTGAAGCG
*pykF*-K319Q-R	TTGACCTTTCGCCGTTTCACCAGAAAG
*pykF*-K319R-F	AAACGGCGAAAGGTAGATACCCTGTT
*pykF*-K319R-R	CTACCTTTCGCCGTTTCACCAGAAA
*pykF*-K340Q-F	ACTGACTCAGCGCTACAAGCTGAACTAGGTTCT
*pykF*-K340Q-R	TTGTAGCGCTGAGTCAGTACGGTTCGC
*pykF*-K340R-F	CTGACTCAGCGCTAAGAGCTGAACTA
*pykF*-K340R-R	CTTAGCGCTGAGTCAGTACGGTTCG
*pykF*-K368Q-F	TAGACACAGCTGAGCAACTAGCTGCTCCACTT
*pykF*-K368Q-R	TTGCTCAGCTGTGTCTACTGCACCTTT
*pykF*-K368R-F	TAGACACAGCTGAGAGACTAGCTGCT
*pykF*-K368R-R	CTCTCAGCTGTGTCTACTGCACCTTT
*pykF*-K382Q-F	GCAACTGAAGGCGGTCAGTCTGCACGTTCAGTA
*pykF*-K382Q-R	CTGACCGCCTTCAGTTGCAACAACGA
*pykF*-K382R-F	CAACTGAAGGCGGTAGGTCTGCACGT
*pykF*-K382R-R	CTACCGCCTTCAGTTGCAACAACGATA
*pykF*-PMMB-F	CGGGGTACCATGAAAAAGACCAAAATCGTATG
*pykF*-PMMB-R-1	GTGGTGGTGGTGGTGTAGTACGTGTACAGAAGCTG
*pykF*-PMMB-R-2	CCCAAGCTTTTAGTGGTGGTGGTGGTGGTGTAGTACGTGTACAGAAGCTG

### Determining PykF Acetylation Sites by Mass Spectrometry

The protocol of LS-MS/MS analysis was performed by following the protocol described in previous studies (38, 39). Purified PykF proteins were fractionated on a 4–20% SDS-PAGE gel. For in-gel tryptic digestion, gel pieces were destained in 50 mM/L NH_4_HCO_3_ in 50% acetonitrile (v/v) until clear. Gel pieces were dehydrated with 100 μl of 100% acetonitrile for 5 min, the liquid removed, and the gel pieces rehydrated in 10 mM/L dithiothreitol and incubated at 56°C for 60 min. Gel pieces were again dehydrated in 100% acetonitrile, liquid was removed and gel pieces were rehydrated with 55 mM/L iodoacetamide. Samples were incubated at room temperature, in the dark for 45 min. Gel pieces were washed with 50 mM/L NH_4_HCO_3_ and dehydrated with 100% acetonitrile. Gel pieces were rehydrated with 10 ng/μl trypsin resuspended in 50 mM/L NH_4_HCO_3_ on ice for 1 h. Excess liquid was removed and gel pieces were digested with trypsin at 37°C overnight. Peptides were extracted with 50% acetonitrile /5% formic acid, followed by 100% acetonitrile. Peptides were dried to completion and resuspended in 2% acetonitrile/0.1% formic acid.

The tryptic peptides were dissolved in 0.1% formic acid (solvent A), directly loaded onto a home-made reversed-phase analytical column (15-cm length, 75 μm i.d.). The gradient was comprised of an increase from 7 to 25% solvent B (0.1% formic acid in 98% acetonitrile) over 18 min, 25 to 38% in 6 min and climbing to 80% in 3 min then holding at 80% for the last 3 min, all at a constant flow rate of 450 nl/min on an EASY-nLC 1000 UPLC system.

The peptides were subjected to NSI source followed by tandem mass spectrometry (MS/MS) in OrbitrapFusion (Thermo) coupled online to the UPLC. The electrospray voltage applied was 2.0 kV. The m/z scan range was 350 to 1,550 for full scan, and intact peptides were detected in the Orbitrap at a resolution of 60,000. Peptides were then selected for MS/MS using NCE setting as 35 and the fragments were detected in the Orbitrap at a resolution of 15,000. A data-dependent procedure that alternated between one MS scan followed by 20 MS/MS scans with 15.0 s dynamic exclusion. Automatic gain control (AGC) was set at 5E4. The peptides eluted from the HPLC column/electrospray source were subjected to MS survey scans. Raw MS/MS data were used to search a user-defined amino acid sequence database with the Proteome Discoverer 1.3 program. Cysteine Alkylation was used as a fixed modification, while lysine acetylation was set as the variable modification.

### Expression and Purification of Lysine Deacetylated PykF Variants

The gene of *pykF* and their variants were cloned into the pET-28a plasmid with 6 xHis-tag and transformed into BL21 (DE3) cells (CD601, TransGen Biotech, Beijing, China) for expression and purified using BeyoGold™ His-tag Purification Resin (P2218, Beyotime, Shanghai, China) according to the manufacturer's recommended procedures. Site-directed mutagenesis of the *pykF* was performed using a Fast MultiSite Mutagenesis System kit (FM201-01, TransGen Biotech, Beijing, China) following manufacturer's protocol.

### Construction of Deletion Mutants and Site-Directed Mutagenesis Complemented Strains

According to previous studies (40, 41), all deletion mutants and complemented strains were made. Primer pairs used for plasmid construction were included in Table 2. Overlap extension PCR was applied to generate an in-frame deletion of the *pykF* gene on the *V. alginolyticus* wild-type HY9901 chromosome. Two about 500 bp PCR fragments corresponding to genomic sequences flanking *pykF* for chromosomal in-frame deletions. Chromosomal in-frame deletions were cloned into a suicide plasmid (pLP12) by using standard cloning procedures followed by DNA sequencing. The resulting constructs were individually transformed into *E. coli* S17-1 λpir and introduced by conjugation into *V. alginolyticus*. Deletion mutants were selected on 10% sucrose TSA plates. Its presence was subsequently confirmed by PCR using primers located inside of the deleted sequence and subsequent sequencing of the PCR product.

For the construction experiments of the complemented strain, the *pykF* and its variants were cloned into expression vector pMMB207, which incorporates a C-terminal His-tag by PCR. The Fast MultiSite Mutagenesis System kit was used to perform site-directed mutagenesis of *pykF* with the corresponding primers (Table 2). These constructs were fully sequenced to check their inserts and then introduced by conjugation into the appropriate mutant strains. Then, all the resulting target mutations were confirmed by DNA sequencing. In addition to this, His Tag Mouse Monoclonal Antibody was used to confirm the normal expression by Western blot (described below).

### PykF Activity Evaluation

Purified PykF and its variants were quantified using the detergent compatible Bradford protein assay kit (P0006, Beyotime, Shanghai, China). Pyruvate kinase enzymatic activity was measured using the pyruvate kinase Activity Detection Kit (BC0540, Solarbio, Beijing, China) according to the manufacturer's instruction. Pyruvate kinase catalyzes phosphoenolpyruvate and ADP to generate ATP and pyruvate, and lactate dehydrogenase further catalyzes NADH and pyruvate to generate lactate and NAD^+^. The decrease rate of NADH can be measured at OD340nm to reflect PykF activity.

### PykF Deacetylation

For *in vitro* tests, the protocol of the deacetylation assay was performed by following described in previous studies (22–24), the reaction of the deacetylation was performed in the buffer containing 50 mM HEPES (pH 7.0), 5 mM MgCl_2_, 1 mM NAD^+^, 1 mM DTT, and 10% glycerol. The deacetylation reaction was initiated by mixing 5 μg PykF, 5 μg CobB proteins in a total volume of 100 μL and incubated at 37°C for 1 h. The acetylation level of the treated proteins was analyzed by Western blot (described below).

For *in vivo* tests, the acetylation level of the native PykF purified from the WT:*pykF* or Δ*cobB*:*pykF* of *V. alginolyticus* grown in TSB medium was determined by Western blot (described below).

### PykF Acetylation

For *in vitro* tests, the protocol of the acetylation assay was performed by following described in previous studies (22–24), the reaction of the acetylation was performed in the buffer containing 50 mM HEPES (pH 7.0), 0.1 mM EDTA, 10% glycerol, 1 mM DTT, and 10 mM sodium butyrate. The acetylation reaction was initiated by mixing 5 μg PykF varied concentrations of AcP in a total volume of 100 μL and then incubated at 37°C for 1 h. The acetylation level of the treated proteins was analyzed by Western blot (described below).

### Determination of Growth and Extracellular Protease Activity

The method of detection of growth was performed by following described in previous studies (41). All strains were incubated separately in TSB overnight and all diluted to (OD_595_ = 0.2), then inoculated at 28°C at a ratio of 1:100 and determined OD_595_ every hour. All measurements were repeated three times per group.

The method of detection of extracellular protease activity was performed by following described in previous studies (41). All strains were coated on TSA plates coated with sterile cellophane, and cultured at 28°C at 24 h, washed with sterile cooled PBS, centrifuged at 4°C for 30 min, and the supernatant filtered to obtain extracellular products. All measurements were repeated three times per group.

### LD_50_ Assessment

The LD_50_ evaluation was performed by following described in previous studies (41). The injection concentrations used for the dose response of wild-type strain HY9901, Δ*pykF*, and all complemented strains were 10^4^, 10^5^, 10^6^, 10^7^, and 10^8^ CFU/mL. A total of 930 fish were randomly divided into seven groups (Table 3). The water temperature was adjusted to 28°C. The experiment was repeated three times. Five microliter of bacterial solution was injected into fish by intramuscular injection. The control group was injected with equivalent volumes of PBS. Fish were monitored for 14 days or until no morbidities occurred.

**Table 3:** Experiment of LD_50_.

**Concentration (CFU/mL)**	**10^**8**^**	**10^**7**^**	**10^**6**^**	**10^**5**^**	**10^**4**^**	**0 (PBS)**
WT	10 ×3	10 ×3	10 ×3	10 ×3	10 ×3	–
Death rate (%)	90	80	60	60	40	–
Δ*pykF*	10 ×3	10 ×3	10 ×3	10 ×3	10 ×3	–
Death rate (%)	66.7	60	46.7	26.7	20	–
Control (PBS)	–	–	–	–	–	10 ×3
Death rate (%)	–	–	–	–	–	–
Δ*pykF:pykF*	10 ×3	10 ×3	10 ×3	10 ×3	10 ×3	–
Death rate (%)	90	70	66.7	60	40	–
Δ*pykF:*K52R	10 ×3	10 ×3	10 ×3	10 ×3	10 ×3	–
Death rate (%)	76.7	66.7	53.3	26.7	23.3	–
Δ*pykF:*K68R	10 ×3	10 ×3	10 ×3	10 ×3	10 ×3	–
Death rate (%)	73.3	63.3	50	26.7	20	–
Δ*pykF:*K317R	10 ×3	10 ×3	10 ×3	10 ×3	10 ×3	–
Death rate (%)	80	70	53.3	30	30	–

### Western Blot

Protein concentrations were determined by Bradford Protein Assay (P0006C, Beyotime, Shanghai, China). For Western blot, purified PykF and its variants were fractionated on a 4–20% SDS-PAGE gel and transferred to the PVDF membrane by Trans-Blot Turbo (Bio-rad, USA). The samples were blocked with QuickBlock™ Blocking Buffer for Western blot (P0239, Beyotime, Shanghai, China) overnight at 4°C and then incubated with the horseradish peroxidase (HRP)-conjugated acetyl lysine antibody (9441S, Cell Signaling Technology, USA) as primary antibodies. The solutions were diluted at a ratio of 1: 2000 in QuickBlock™ Primary Antibody Dilution Buffer for Western blot (P0239, Beyotime, Shanghai, China) for 2 h at room temperature. Afterward, the membranes were washed three times in TBST and incubated with HRP-labeled goat anti-rabbit IgG (H+L) (A0208, Beyotime, Shanghai, China) at room temperature for 1 h. Antigen–antibody complexes were detected via the enhanced chemiluminescence method (P0018, Beyotime, Shanghai, China).

### Statistical Analysis

Western blot images were quantified using Image J software. The experimental data were analyzed by single factor analysis of variance (ANOVA) with SPSS17.0 software and GraphPad Prism 8. The results in the figures are displayed as the mean ± standard deviation. Differences were considered statistically significant if the *P*-value was smaller than 0.05. Significance was indicated as ^*^P ≤ 0.05; no ^*^, *P* > 0.05, no significance.

## Results

### The Effect of Lysine Deacetylation and Acetylation Status of PykF on Pyruvate Kinase Activity

The acetylation signal of PykF was detected by Western blot, and then 11 acetylated lysine residues were identified via the previous acetylome profiling of *V. alginolyticus* and the mass spectrometry data (Supplementary Figures 1–11). Subsequently, 11 lysine residues, namely, K13, K19, K52, K59, K68, K145, K317, K319, K340, K368, and K382 were identified and analyzed to uncover the effects of deacetylated lysine residues on PykF.

**Figure 1:**
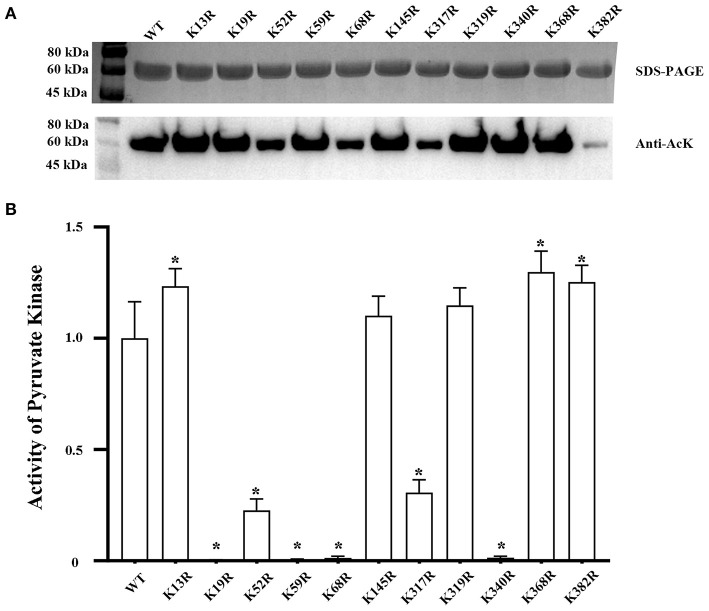
**(A)** Top line, SDS-PAGE and Western blot analysis of purified PykF and its acetylated variants from BL21 (DE3) cells. The acetylated variants with mutation of a single lysine site in PykF to glutamine. Lane 1, wild-type PykF; Lane 2, PykF-K13Q; Lane 3, PykF-K19Q; Lane 4, PykF-K52Q; Lane 5, PykF-K59Q; Lane 6, PykF-K68Q; Lane 7, PykF-145Q; Lane 8, PykF-K317Q; Lane 9, PykF-K319Q; Lane 10, PykF-K340Q; Lane 11, PykF-K368Q; Lane 12, PykF-K382Q. The same amount of protein was loaded. Anti-AcK: anti-acetyl lysine antibody. Bottom line, pyruvate kinase activity of PykF and its acetylated variants. Pyruvate kinase activity of the wild-type PykF was set as 1. Mean values and standard deviations were calculated based on three replicates (*n* = 3). **(B)** Top line, SDS-PAGE and Western blot analysis of purified PykF and its deacetylated variants from BL21 (DE3) cells. The deacetylated variants with mutation of a single lysine site in PykF to arginine. Lane 1, wild-type PykF; Lane 2, PykF-K13R; Lane 3, PykF-K19R; Lane 4, PykF-K52R; Lane 5, PykF-K59R; Lane 6, PykF-K68R; Lane 7, PykF-145R; Lane 8, PykF-K317R; Lane 9, PykF-K319R; Lane 10, PykF-K340R; Lane 11, PykF-K368R; Lane 12, PykF-K382R. The same amount of protein was loaded. Anti-AcK, anti-acetyl lysine antibody. Bottom line, pyruvate kinase activity of PykF and its deacetylated variants. Pyruvate kinase activity of the wild-type PykF was set as 1. Mean values and standard deviations were calculated based on three replicates (*n* = 3). Two-tailed *P*-values were determined by the *t*-test, and the significance level is 0.05. *p < 0.05.

Acetylation levels of PykF and its acetylation and deacetylated variants were evaluated. Compared to the acetylation levels of PykF, the acetylation mimicking status of those sites were not significantly different from PykF (Figure 1A top line). But the effect of deacetylation at different sites on acetylation level was different. Compared to the acetylation levels of PykF, the deacetylation mimicking status of K52, K68, K317, and K382 significantly decreased acetylation level, but other deacetylation mimicking status were no significant difference (Figure 1B top line).

Pyruvate kinase activities of PykF and its acetylation and deacetylated variants were evaluated. Compared to pyruvate kinase activities of PykF, the acetylation mimicking status of those sites were not significantly different from PykF (Figure 1A bottom line). But the effect of deacetylation at different sites on pyruvate kinase activities was different. Compared to the pyruvate kinase activities of PykF, the deacetylation mimicking status of K13, K368, or K382 significantly increased the activity, while acetylation at K145 or K319 only had no significantly change. Furthermore, the deacetylation mimicking status of K52 or K317 lost about 80% of pyruvate kinase activity, while the deacetylation mimicking status of K19, K59, K68, or K340 almost eliminated its activity (Figure 1B bottom line).

### PykF Activity of the Deletion Mutants and Site-Directed Mutagenesis Complemented Strains

**Figure 2:**
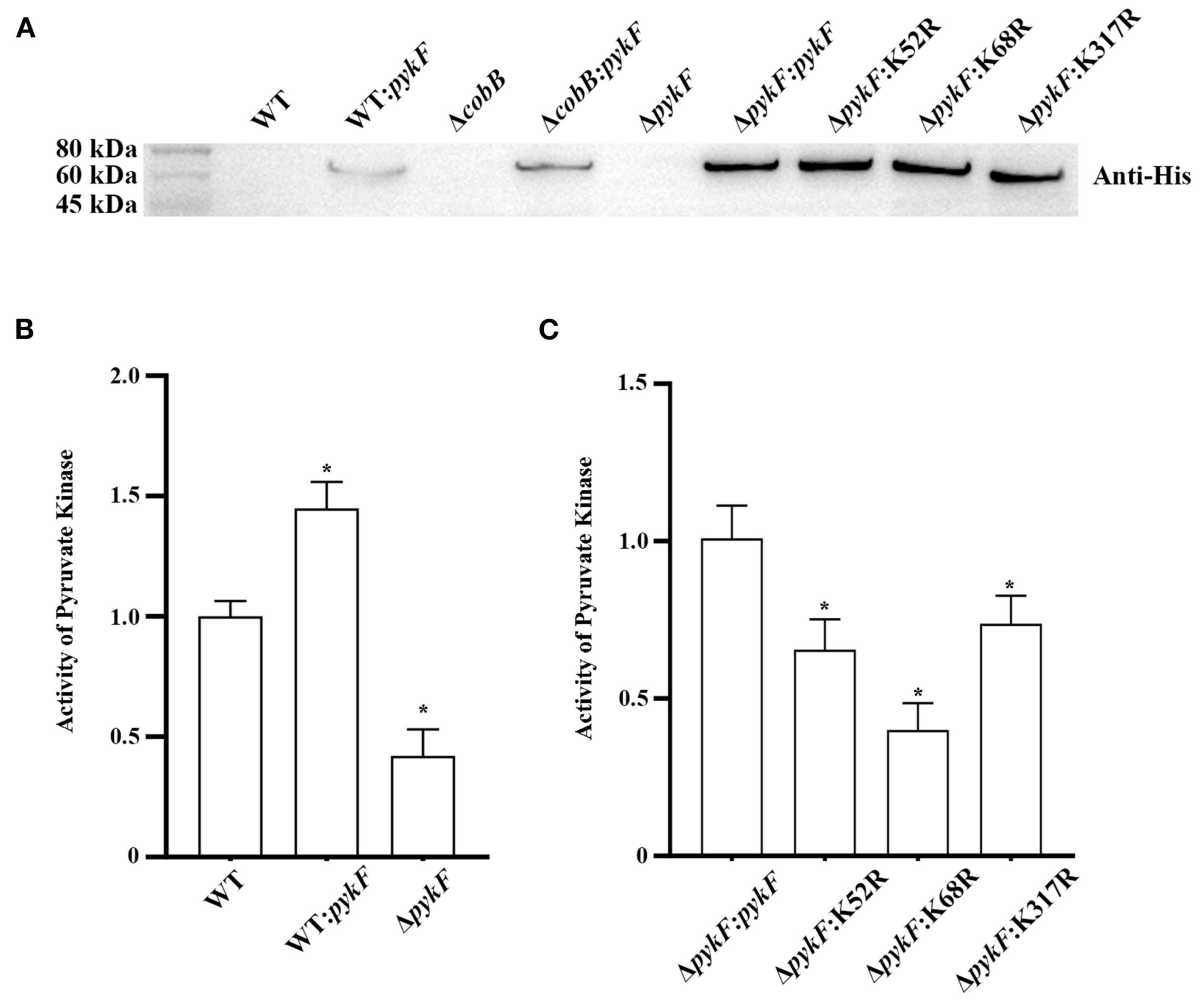
**(A)** Construction of complemented and overexpression strains. Lane 1, wild-type (WT); Lane 2, WT:*pykF* overexpression strain; Lane 3, deletion of *cobB* gene strain (Δ*cobB*); Lane 4, Δ*cobB*:*pykF* complemented strain; Lane 5, deletion of *pykF* gene strain (Δ*pykF*); Lane 6, Δ*pykF:pykF* complemented strain; Lane 7, Δ*pykF*:K52R complemented strain; Lane 8, Δ*pykF*:K68R complemented strain; Lane 9, Δ*pykF*:K317R complemented strain. Anti-His: His Tag Mouse Monoclonal Antibody. **(B)** Analysis of pyruvate kinase activity throughout the *V. alginolyticus* Δ*pykF* strain and WT:*pykF* overexpression strain. Pyruvate kinase activity of *V. alginolyticus* WT was set as 1. **(C)** Analysis pyruvate kinase activity of site-directed mutagenesis complemented strains. Pyruvate kinase activity of *V. alginolyticus* Δ*pykF:pykF* was set as 1. Mean values and standard deviations were calculated based on three replicates (*n* = 3). Two-tailed *P*-values were determined by the *t*-test, and the significance level is 0.05. *p < 0.05.

The principle of lysine site selection for the next experiments was that deacetylated variants had significantly lower acetylation levels and activity than PykF. According to the results 3.1, K52, K68, and K317 were selected to study the changes in pyruvate kinase activity. In this study, WT: *pykF* expression strain, Δ*pykF* mutant strain (Figure 6 of Supplementary Files), Δ*pykF*: K52R, Δ*pykF*: K68R, Δ*pykF*: K317R site-directed mutagenesis strains were successful constructed (Figure 2A). Pyruvate kinase activity of Δ*pykF* was decreased by about 60% compared to wild-type (Figure 2B). Site-directed mutagenesis complemented strains such as Δ*pykF*:K52R, Δ*pykF*:K68R and Δ*pykF*:K317R showed significantly decreased pyruvate kinase activity compared to Δ*pykF:pykF* complemented strain (Figure 2C). Therefore, the deacetylation status of K52, K68, and K317 was required for pyruvate kinase activity of *V. alginolyticus*.

### Deacetylation of PykF by Deacetylase CobB

**Figure 3:**
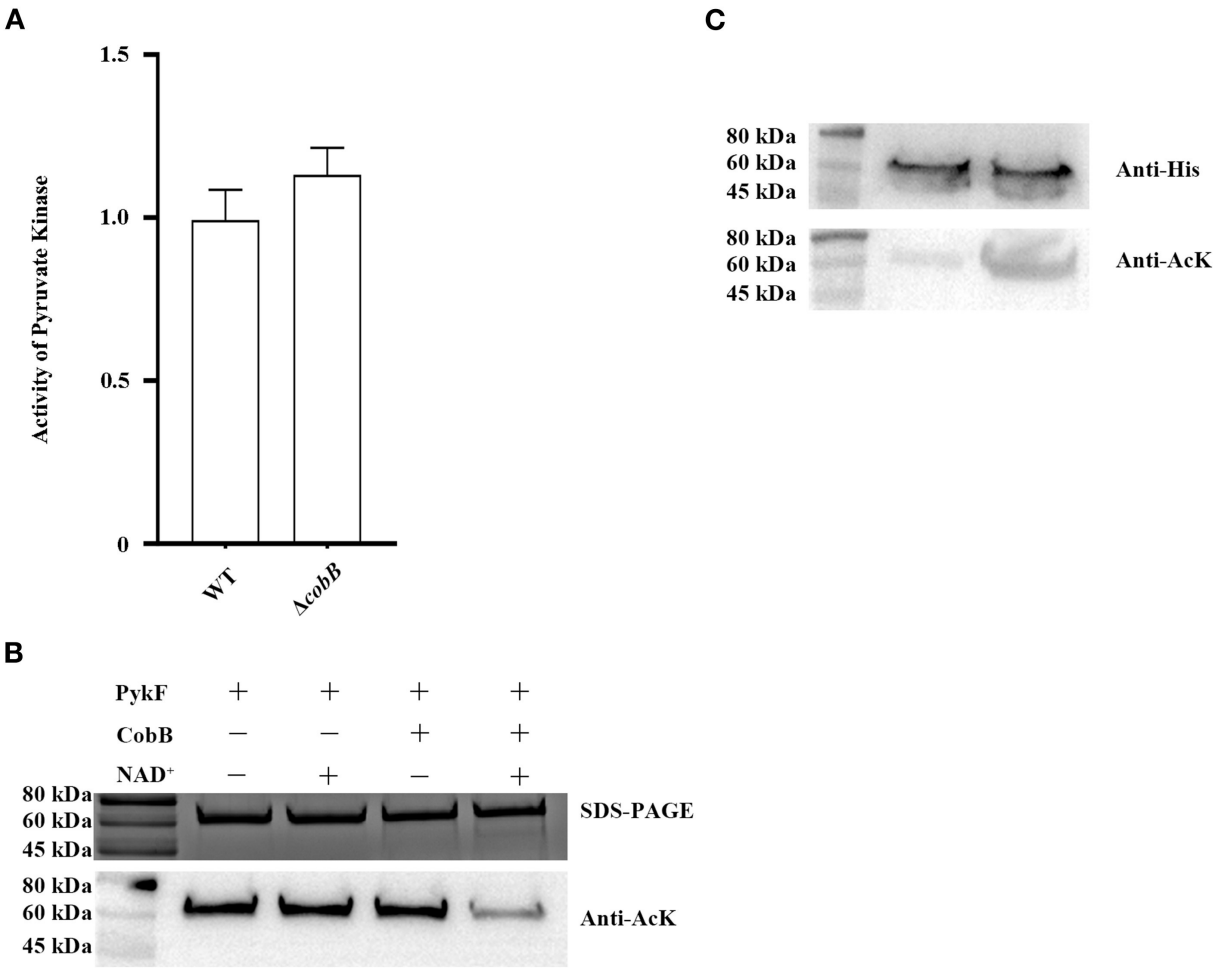
**(A)** CobB deacetylation PykF *in vitro*. Top line, Western blot was performed on PykF treated with CobB for 1 h. The same amount of protein was loaded in all lanes. Bottom line, pyruvate kinase activity was measured, and the enzyme activity of lane 1 sample was set as 1. Mean values and standard deviations were calculated based on three replicates (*n* = 3). Two-tailed *P*-values were determined by the *t*-test, and the significance level is 0.05. **(B)** CobB deacetylates PykF *in vivo*. Top line, Western blot analysis of purified PykF from *V. alginolyticus* WT:*pykF* and Δ*cobB*:*pykF*. Lane 1, purified PykF from *V. alginolyticus* WT:*pykF*; Lane 2, purified PykF from *V. alginolyticus* Δ*cobB*:*pykF*. The same amount of protein was loaded in all lanes. Anti-His, His Tag Mouse Monoclonal Antibody. Anti-AcK: anti-acetyl lysine antibody. Bottom line, pyruvate kinase activity of *V. alginolyticus* WT and Δ*cobB*. Pyruvate kinase activity of *V. alginolyticus* WT was set as 1. Mean values and standard deviations were calculated based on three replicates (*n* = 3). Two-tailed *P*-values were determined by the *t*-test, and the significance level is 0.05.

The acetyl on the ε-amino group of lysine residues is stable, and its removal needs a category of enzymes called lysine deacetylases (42). So far, only one lysine deacetylase has been identified in bacteria: the deacetylase CobB, a NAD^+^-dependent sirtuin class deacetylase (43, 44). we have verified the function of the CobB protein in *V. alginolyticus* (45). To confirm the effect of CobB on PykF, Western blot was used to detect the deacetylation of CobB to PykF, and then its enzyme activity was determined. The results showed that the CobB expressed and purified from *E.coli* BL21 (DE3) cells can deacetylate PykF with the participation of NAD^+^ (Figure 3A top line), and deacetylation of PykF significantly enhanced pyruvate kinase activity (Figure 3A bottom line). For *in vivo* tests, the acetylation level of the native PykF purified from the WT:*pykF* or Δ*cobB*:*pykF* was determined by Western blot. Deletion of the *cobB* gene increased the acetylation level of PykF (Figure 3B top line), but it was no significant difference in pyruvate kinase activity (Figure 3B bottom line). In conclusion, CobB can regulate the acetylation level of PykF and pyruvate kinase activity.

### Acetylation of PykF by Acetyl Phosphate

**Figure 4:**
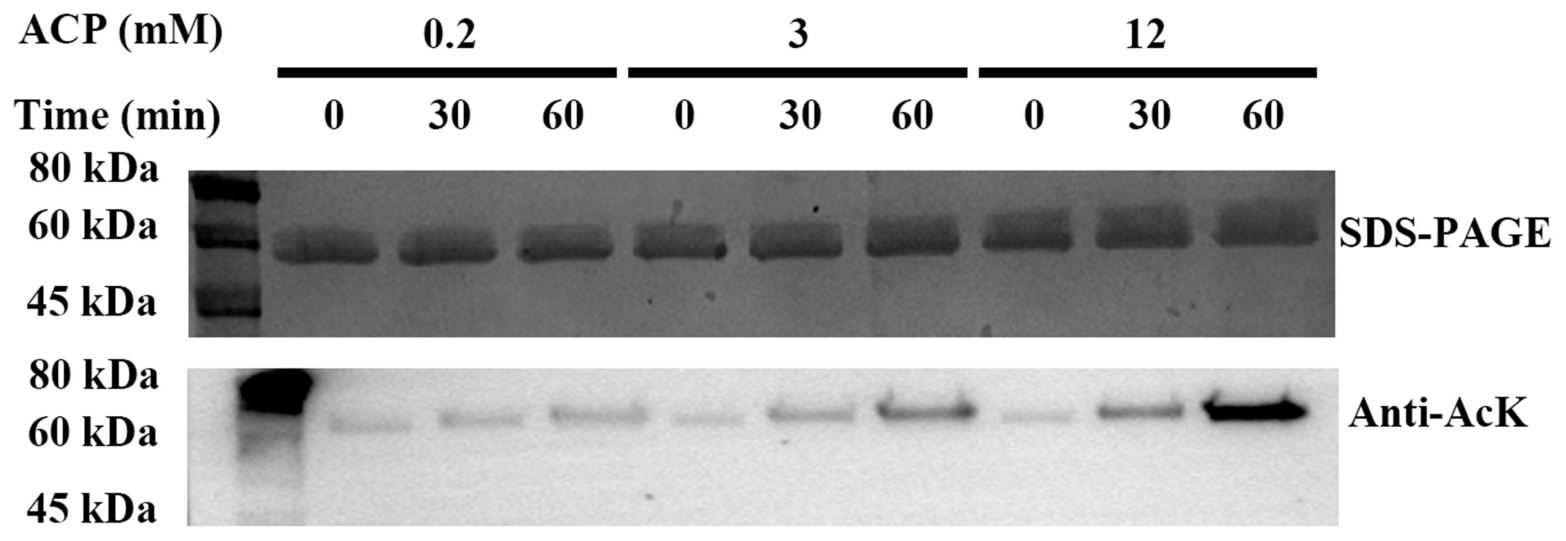
Top line, acetylation of PykF. SDS-PAGE and Western blot analysis were performed on purified PykF treated with AcP in different concentrations and incubation time. The same amount of protein was loaded. Anti-AcK, acetyl lysine antibody. Bottom line, pyruvate kinase activity was measured, and the enzyme activity of lane 1 sample was set as 1. Mean values and standard deviations were calculated based on three replicates (*n* = 3). Two-tailed *P*-values were determined by the *t*-test, and the significance level is 0.05.

So far, it is believed that there are two mechanisms of lysine acetylation in bacteria, the acetyl-CoA-dependent enzymatic process, and the AcP-dependent chemical reaction (11, 12, 14, 31, 46, 47). In order to study the acetylation mechanism of PykF, PykF expressed and purified from BL21 (DE3) cells was treated with AcP at concentrations of 200 μM, 3 mM, and 12 mM, corresponding to estimated intracellular AcP concentrations at the exponential phase, the stationary phase, and the ΔackA background, which accumulates AcP, respectively (30, 48, 49). The immunoblot with anti-AcK antibody and the detection of pyruvate kinase activity indicated that AcP can chemically acetylate PykF in a dose-dependent and time-dependent manner *in vitro* (Figure 4 top line). Furthermore, with the increase of incubation time, the acetylation level of PykF increased and its enzyme activity decreased correspondingly (Figure 4 bottom line). This suggested AcP can acetylate PykF, resulting in a decrease in enzyme activity of PykF.

### Growth and Extracellular Protease Activity

**Figure 5:**
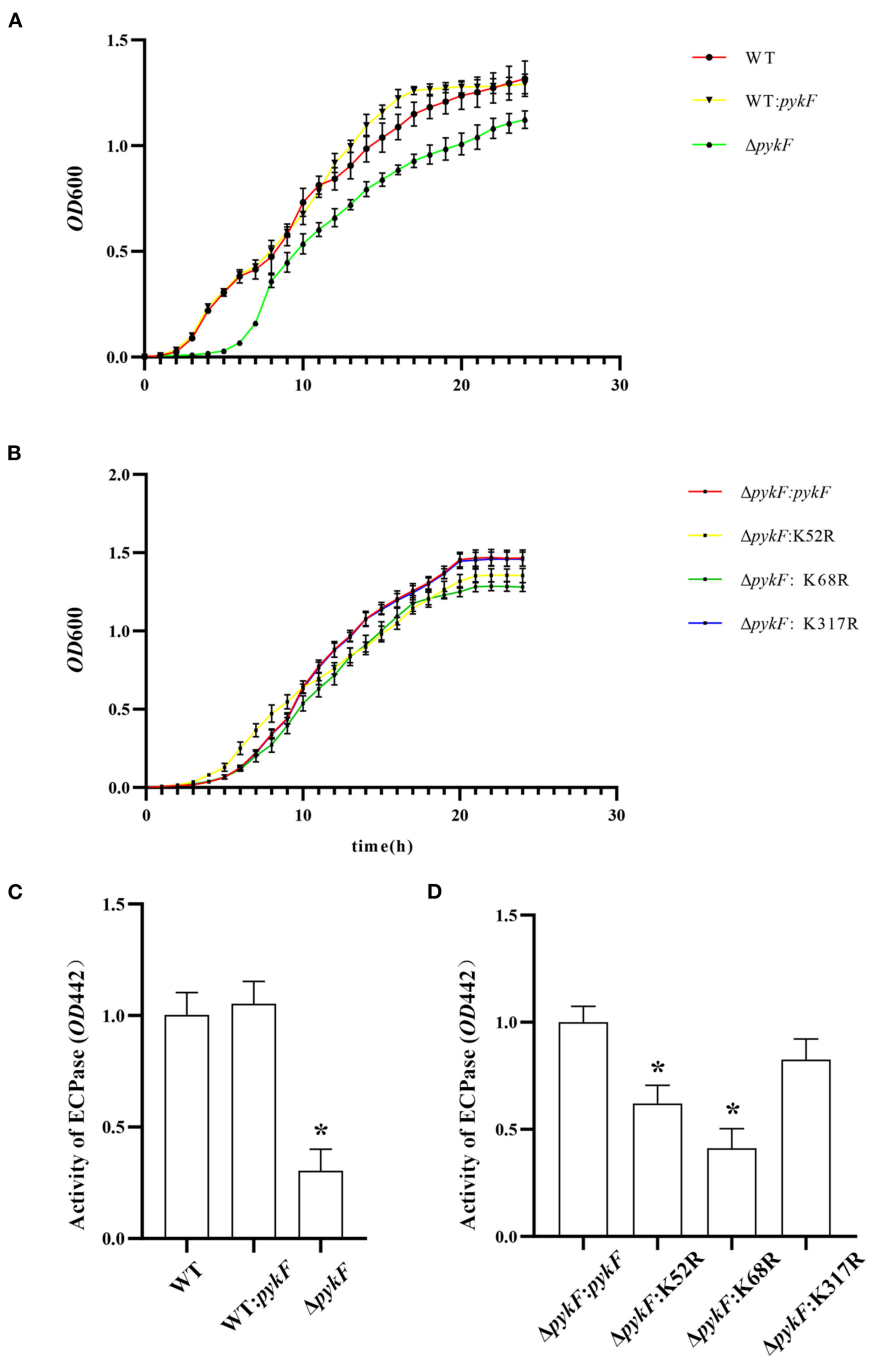
**(A)** Growth of *V. alginolyticus*. Analysis of growth throughout the *V. alginolyticus* Δ*pykF* strain and WT:*pykF* overexpression strain at 24 h. **(B)** Analysis of growth throughout *V. alginolyticus* Δ*pykF*:*pykF*, Δ*pykF*:K52R, Δ*pykF*: K68R and Δ*pykF*:K317R complemented strains at 24 h. **(C)** Analysis of extracellular protease activity throughout the *V. alginolyticus* Δ*pykF* mutant strain and WT:*pykF* overexpression strain. Extracellular protease activity of *V. alginolyticus* WT was set as 1. **(D)** Analysis site-directed mutagenesis complemented strains on extracellular protease activity. Extracellular protease activity of *V. alginolyticus* Δ*pykF:pykF* was set as 1. Mean values and standard deviations were calculated based on three replicates (*n* = 3). Two-tailed *P*-values were determined by the *t*-test, and the significance level is 0.05. *p < 0.05.

To determine the effect of delete or overexpress *pykF* gene and site-directed mutagenesis at the K7, K52, and K317 on the biological function of *V. alginolyticus*. WT, Δ*pykF* mutant strain, and WT:*pykF*, Δ*pykF:pykF* complemented strain, and Δ*pykF*:K52R, Δ*pykF*:K68R and Δ*pykF*:K317R site-directed mutagenesis complemented strains were subjected to determine growth and extracellular protease activity. The results showed that the growth rate of the WT:*pykF* overexpression strain was similar to that of the wild-type strain, while the growth rate of the Δ*pykF* mutant strain was significantly reduced (Figure 5A). In addition, the growth rate of site-directed mutagenesis complemented strains had no significant change compared to the Δ*pykF*:*pykF* complemented strain (Figure 5B). These results indicate that deletion of *pykF* gene significantly weakened the growth of *V. alginolyticus*, whereas overexpression of *pykF* gene and site-directed mutagenesis complemented strains had no significant effect on the growth of *V. alginolyticus*.

Extracellular protease, as metabolites of bacteria, play an important role as virulence factors in the process of infecting the host. Extracellular proteins have a variety of protease activities, including lecithin, amylase, lipase, and casein. The extracellular protease activity was reduced in the Δ*pykF* mutant strain compared to the wild-type strain (Figure 5C). Compared to the Δ*pykF*:*pykF* complemented strain, site-directed mutagenesis complemented strains Δ*pykF*:K52R and Δ*pykF*:K68R showed decreased extracellular protease activity, but extracellular protease activity of Δ*pykF*:K317R had no significant change (Figure 5D). Among them, site-directed mutagenesis complemented strains Δ*pykF*:K52R and Δ*pykF*:K68R showed about a 50% reduction in extracellular protease activity, indicating that deacetylation of these two sites has an important role in protease activity inhibition.

### LD_50_

Zebrafish from quarantined stocks recognized as disease-free were used as models to assess the virulence of WT, Δ*pykF*, and all complemented strains. The results showed that LD_50_ of Δ*pykF* was 6 times higher than that of WT strain. Compared with Δ*pykF*: *pykF* complementary strains, Δ*pykF*: K52R and Δ*pykF*: K68R had about 3 and 4 times of LD_50_, while Δ*pykF*: K317R had no significant change (Tables 3, 4). The results showed that the deletion of *pykF* gene weakened the virulence of *V. alginolyticus*, and the deacetylation of K52 and K68 sites also weakened the virulence of *V. alginolyticus*, while the deacetylation of K317R did not significantly affect the virulence of *V. alginolyticus*.

**Table 4:** Comparison of LD_50_ between WT, Δ*pykF*, and all complemented strains.

**Strain**	**LD_**50**_ (CFU/mL)**
WT	5.0 ×10^5^
Δ*pykF*	3.2 ×10^6^
Δ*pykF:pykF*	4.6 ×10^5^
Δ*pykF:*K52R	1.1 ×10^6^
Δ*pykF:*K68R	1.8 ×10^6^
Δ*pykF:*K317R	6.0 ×10^5^

*Values are mean ± standard deviation for three trials*.

## Discussion

In recent years, more and more acetylome data have been published, showing that most acetylated proteins are mostly involved in cellular metabolism. However, no studies have been reported the effect of acetylation modifications on PykF in bacteria (13, 27, 50–52). In this study, we show the effect of deacetylation of lysine residues on PykF, and that deacetylases CobB can deacetylate PykF. As a classic approach, glutamine is commonly used as a mimic of acetylated lysine. Inconsistent with previous research is that PykF expressed in *E.coli* BL21 (DE3) cells had been detected acetylation signal (22–24). Glutamine was used to substitute for lysine to mimic acetylation, but acetylation mimicking status of those sites cannot significantly change their acetylation levels and pyruvate kinase activity. The reason for this may be that those lysine residues were already acetylated in prokaryotic expression system. On the other hand, arginine is commonly used as a mimic of deacetylated lysine, and then we used Q5 site-directed mutagenesis kit to mutate acetylated lysine residues to arginine, and measured pyruvate kinase activity of these variants to mimic the effect of lysine deacetylation on its activity. By site-specifically deacetylated from selected acetylated lysine of PykF, we provided a direct biochemical basis for the deacetylation study on PykF. We found that deacetylation status of K52, K68, and K317 sites decreased pyruvate kinase activity. Thus, the acetylation of these three lysine residues is essential for its activity.

CobB is a bacterial Sirtuins that regulates the function of its substrate by deacetylation at the active site of lysine. Although CobB is a predominant deacetylase in bacteria, it does not completely deacetylate PykF due to CobB only deacetylates a portion of acetyl lysine (30, 43). Our results showed that PykF can be deacetylated by the NAD^+^-dependent deacetylase CobB *in vitro* and that hyperacetylation of PykF occurs in the Δ*cobB* strain. These suggest that CobB can also regulate the deacetylation of PykF *in vivo*. We further found that pyruvate kinase activity has no significant difference in the Δ*cobB* strain compared to the wild-type strain. But in other studies, deletion of *cobB* gene increases the activity of some metabolic enzymes from other strains. CobB affects malate dehydrogenase activity by regulating the deacetylation of lysine residues at positions K301 and K314 of the non-protein structural domain of *E. coli* Malate Dehydrogenase (22). This regulatory mechanism has been more studied in eukaryotes, Sirtuin family deacetylation mediates nuclear localization of PKM2, protein kinase activity of pyruvate kinase M2, tetramerization and pyruvate kinase activity *in vitro* to influence oncogenic function, tumor growth and insect longevity (53–58). This indicates the importance of deacetylation for the regulation of PykF function, and the preference of CobB for acetyl groups may be a new ideal drug target.

Not all acetylations are reversed, and we obtained a protein that all the lysine sites may not have been acetylated. We found that PykF was acetylated *in vitro* in a time- and AcP dose-dependent manner, and with the increase of acetylation level, its enzyme activity decreases. This suggesting that there are still unidentified PykF acetylated lysine residues, or the known sites may be more acetylated. This has similarities with the studies in *E. coli* (22–24). Preliminary findings in *E. coli* revealed that most acetylation occurred at a low level and accumulated in growth-arrested cells in a manner that depended on the formation of AcP through glycolysis (30). The site-specificity of AcP-dependent protein acetylation has been studied, and these specificities depend on the surface accessibility, reactivity, and three-dimensional microenvironment of the target lysine. AcP affects the function of some key enzymes of bacteria as well as mediating bacterial virulence (15, 31, 59, 60). The study of AcP-dependent acetylation of substrates is important for understanding the mechanism of AcP action.

K52, K68, and K317 sites of *V.alginolyticus* were mutated to arginine to mimic the effect of deacetylation status on it. We verified the acetylation levels by Western blot and found that protein acetylation levels were reduced in mutation at K52, K68, and K317 sites, and the three site-directed mutagenesis complemented strains showed decreased pyruvate kinase activity. Lysine deacetylation at the K52 and K68 sites significantly reduced extracellular protease activity of *V. alginolyticus*. But lysine deacetylation at the K317 site had no significant effect on extracellular protease activity. Thus, protein deacetylation modification at these three sites has important effects. An important gene for carbon metabolism-related pyruvate kinase is encoded by *pykF* in many bacteria. In the present study, deletion of the *pykF* gene reduced the growth rate and extracellular protein activity of bacteria, which is consistent with the findings in other bacteria (61–64). However, overexpression of *pykF* gene had no significant effect on the growth rate and extracellular protease activity. We also found decreased rate of extracellular protein activity in complemented strains at the K52R and K68R sites. The two lysine residues are located in the A-domain of PykF, which consists of a (β/α)8-barrel structure characterized by three helices located at the top of the loop connecting the c-terminal chains of the β sheet, and these helices play a major role in catalytic and metastable regulation. The catalytic site of PyK is in the cleft between the A- and B-domains at the top of the barrel (65–69). These effects may be explained by that the deacetylation of the three sites changed the charged nature of the three lysine residues, the hydrophilicity, and the distance between the hydrogen bonds, thus affected the stability of the catalytic domain and the active site.

PykF is one of the key enzymes in glycolysis and plays an important role in *V. alginolyticus* virulence. Our results are like those of other pathogens, such as Brucella abortus (62). The deacetylation of K52 and K68 of PykF is virulent by reducing pyruvate kinase activity and extracellular protease activity of *V. alginolyticus*, but no similar studies have been conducted before. During the entry of intracellular pathogens into host cells, carbon metabolism may be directly or indirectly involved in regulating the expression of virulence genes in host cells, thus affecting the virulence of pathogens. In *V. alginolyticus*, silencing of the *pykF* gene reduces the expression level of some virulence genes, *ndk* (Nucleoside-diphosphate kinase encoding gene), *eno* (Enolase encoding gene), *sdhB* (succinate dehydrogenase iron-sulfur Subunit encoding Gene), *glpF* (glycerol uptake facilitator Protein-encoding gene) and *cycH* (phosphoadenosine phosphosulfate reductase encoding gene) (64), which in turn leads to reduced virulence. The deletion of *pykF* gene also showed a consistent situation, which proved that *pykF* is closely related to the regulation of virulence of *V. alginolyticus*.

## Conclusion

In this study, we investigated the effect of lysine deacetylation of PykF protein on its biological function. The results showed that deacetylation status of three lysine residues in PykF, K52, K68, and K317, significantly reduced its activity. Deacetylated at the K52 and K68 sites significantly reduced extracellular protease activity and virulence of *V. alginolyticus*, but deacetylated at the K317 site had no significant difference on extracellular protease activity. Deacetylase CobB deacetylates PykF, and AcP catalyzes the acetylation of PykF. And with the acetylation level of PykF increased and its enzyme activity decreased. In summary, although deacetylation status of three lysine residues, K52, K68, and K317, all reduced pyruvate kinase activity. However, the mechanisms of acetylation and deacetylation of these sites are not clear and need to be further investigated. We are sure that the deacetylation modifications of these sites will become new drug targets that can better reduce the risk of vibriosis to the aquaculture industry.

## Data Availability Statement

The datasets presented in this study can be found in online repositories. The names of the repository/repositories and accession number(s) can be found in the article/Supplementary Material.

## Ethics Statement

The animal study was reviewed and approved by Guangli Li and Guangdong Ocean University of Ethics Committee.

## Author Contributions

ZX: conceptualization, methodology, and writing—original draft. LW, XW, MW, and MT: resources and investigation. YD: writing—review and editing and supervision. All authors contributed to the article and approved the submitted version.

## Funding

This work was supported by Foundation for the High-level Talents in Higher Education of Guangdong and Graduate Education Innovation Program of Guangdong Ocean University (No. 201724).

## Conflict of Interest

The authors declare that the research was conducted in the absence of any commercial or financial relationships that could be construed as a potential conflict of interest.

## Publisher's Note

All claims expressed in this article are solely those of the authors and do not necessarily represent those of their affiliated organizations, or those of the publisher, the editors and the reviewers. Any product that may be evaluated in this article, or claim that may be made by its manufacturer, is not guaranteed or endorsed by the publisher.
