# Supplementary figures and images for: Characterizing the Effect of the Lysine Deacetylation Modification on Enzyme Activity of Pyruvate Kinase I and Pathogenicity of Vibrio alginolyticus
Source: Front Vet Sci. 2022 Jun 6;9:877067. doi: 10.3389/fvets.2022.877067 (PMC9252168; doi:10.3389/fvets.2022.877067)

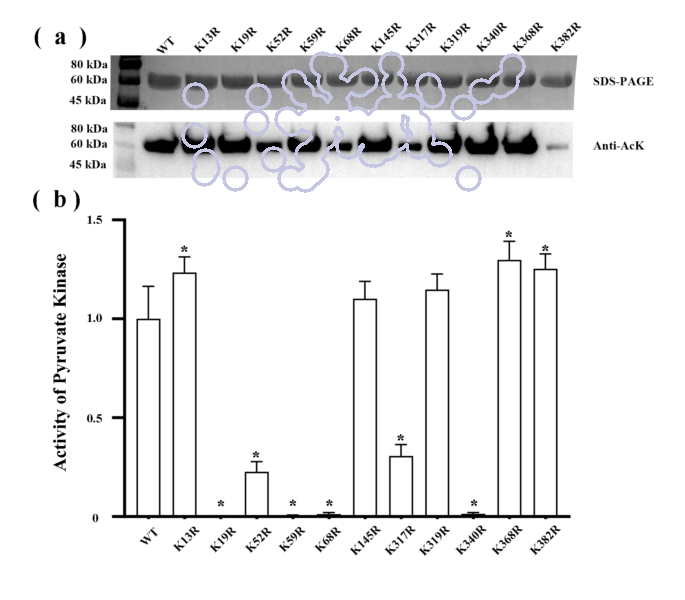

Supplement: Supplementary file 1 [file Image_1.PNG]
