# Supplementary material for: Characterizing the Effect of the Lysine Deacetylation Modification on Enzyme Activity of Pyruvate Kinase I and Pathogenicity of Vibrio alginolyticus
Source: Front Vet Sci. 2022 Jun 6;9:877067. doi: 10.3389/fvets.2022.877067 (PMC9252168; doi:10.3389/fvets.2022.877067)
Supplement: Supplementary file 2 [file Table_1.docx]

Fig.S1 LC-MS/MS analysis of PykF 13-AcK

Fig.S2 LC-MS/MS analysis of PykF 19-AcK

Fig.S3 LC-MS/MS analysis of PykF 52-AcK

Fig.S4 LC-MS/MS analysis of PykF 59-AcK

Fig.S5 LC-MS/MS analysis of PykF 68-AcK

Fig.S6 LC-MS/MS analysis of PykF 145-AcK

Fig.S7 LC-MS/MS analysis of PykF 317-AcK

Fig.S8 LC-MS/MS analysis of PykF 319-AcK

Fig.S9 LC-MS/MS analysis of PykF 340-AcK

Fig.S10 LC-MS/MS analysis of PykF 368-AcK

Fig.S11 LC-MS/MS analysis of PykF 382-AcK

|  |  |  |  |
| --- | --- | --- | --- |
|  |  |  |  |
|  |  |  |  |
|  |  |  |  |
|  |  |  |  |
|  |  |  |  |
|  |  |  |  |
